# Cardiac Transplantation: Update on a Road Less Traveled

**DOI:** 10.31486/toj.19.0022

**Published:** 2019

**Authors:** Tripti Gupta, Selim R. Krim

**Affiliations:** ^1^ Department of Cardiology, John Ochsner Heart and Vascular Institute, Ochsner Clinic Foundation, New Orleans, LA; ^2^ Section of Cardiomyopathy and Heart Transplantation, John Ochsner Heart and Vascular Institute, Ochsner Clinic Foundation, New Orleans, LA; ^3^ The University of Queensland Faculty of Medicine, Ochsner Clinical School, New Orleans, LA

**Keywords:** *Graft rejection*, *heart failure*, *heart transplantation*, *immunosuppression*, *primary graft dysfunction*, *waiting lists*

## Abstract

**Background:** With an aging population, the prevalence of heart failure continues to rise. The use of guideline-directed medical therapy and mechanical circulatory support devices has helped to improve outcomes, but cardiac transplantation remains the definitive treatment for end-stage heart failure.

**Methods:** We provide an update on cardiac transplantation and review indications, contraindications, and important aspects of perioperative and postoperative management. We also highlight the current challenges faced by the transplant community.

**Results:** Advances in surgical techniques and immunosuppression have increased survival rates posttransplant. However, the risk of rejection and adverse effects from chronic immunosuppression continue to affect long-term outcomes.

**Conclusion:** Despite tremendous progress in the management of cardiac transplant patients, we have much opportunity to further optimize cardiac transplant waitlisting and improve posttransplant outcomes.

## INTRODUCTION

With the aging of our population and the epidemics of hypertension, coronary artery disease, and obesity, the incidence and prevalence of heart failure (HF) continue to rise. Currently available guideline-directed medical therapy has helped tremendously to improve symptoms and survival of patients with HF with reduced ejection fraction; however, a large number of patients still reach stage D HF.^1^ Mechanical circulatory support (MCS) has emerged as a viable strategy to bridge patients to cardiac transplantation or as an alternative for patients who are not candidates for transplantation. MCS-related complications such as stroke, pump thrombosis, and suboptimal long-term survival remain a challenge. Therefore, cardiac transplantation remains the gold standard and only cure for stage D HF.^1^ This article provides an update on cardiac transplantation, with discussions of indications, contraindications, and perioperative and postoperative management. We address current challenges faced by the transplant community, discuss the updated United Network for Organ Sharing (UNOS) listing system and its implications, and propose future directions.

## BRIEF HISTORIC OVERVIEW

**Figure 1.**
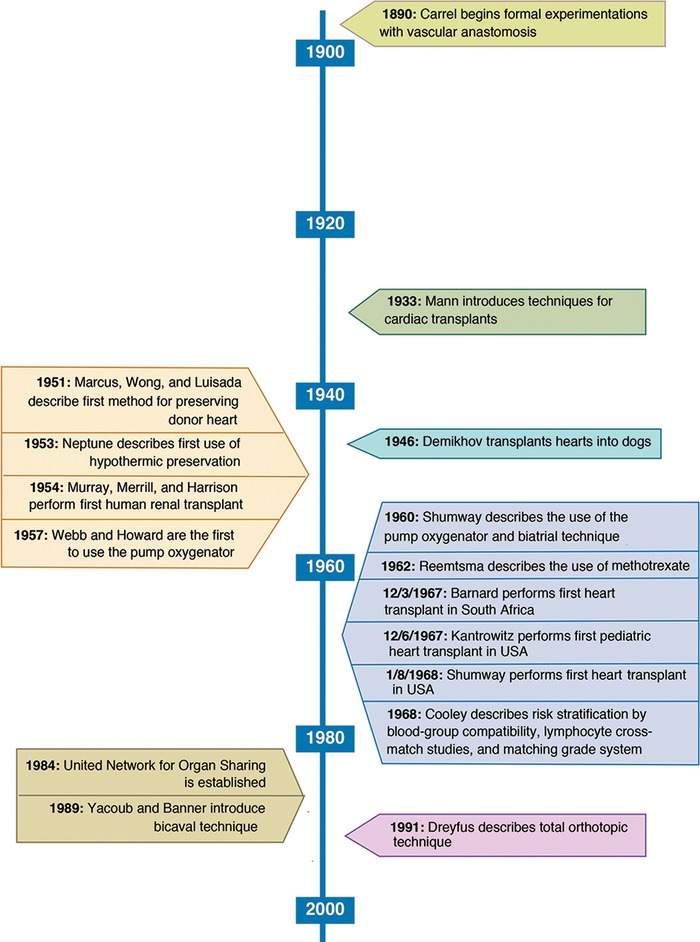
**Advancements in cardiac transplantation.^2^**

Alexis Carrel began experiments with vascular anastomoses in the 1890s, but it wasn’t until 1967 that Dr. Christiaan Barnard successfully completed the first orthotopic cardiac transplantation.^2^ The 54-year-old recipient remained alive for 18 days postoperatively but died of *Pseudomonas* pneumonia.^2^ Inadequate understanding of the rejection process curbed further advancements in cardiac transplantation until Dr. Norman Shumway's discovery in 1978 of cyclosporine A as an immunosuppressant. This breakthrough led to a dramatic improvement in posttransplant outcomes.^2^ Figure 1 shows a timeline of advances in surgical techniques that made cardiac transplantation possible.^2^

## CARDIAC TRANSPLANTATION BY THE NUMBERS

In 2016, 3,209 heart transplants were performed; 2,764 were performed in adult recipients. Between 2005 and 2016, the number of waitlisted patients increased by 57%, but the overall heart transplant rates decreased by 27.8% (129.0 to 93.1 per 100 waitlist years).^3^ Transplant rates have not increased at the same rate as the listings, largely because of a mismatch between demand and supply. The donor rate has not increased and varies by geography. The trend to perform cardiac transplantation in patients >65 years of age, patients with the highest UNOS status, and patients with blood group AB is growing.^3^ Pretransplant mortality on the waitlist decreased from 14.6 deaths per 100 waitlist years in 2015 to 9.7 deaths per 100 waitlist years in 2016.^3^ In a 2014 analysis of cardiac transplantation recipients, the median survival time was approximately 10.7 years, with 84.5% 1-year survival and 72.5% 5-year survival rates.^4^

## INDICATIONS AND CONTRAINDICATIONS FOR CARDIAC TRANSPLANT

The primary indication for cardiac transplantation is persistent advanced chronic HF despite optimal medical management.^5^ Table 1 lists common indications and contraindications for cardiac transplantation proposed by the American College of Cardiology and the American Heart Association.^6-7^ Examples of absolute contraindications are irreversible liver disease or pulmonary parenchymal disease, severe symptomatic cerebrovascular disease, and a history of solid organ or hematologic malignancy. Patients should always be referred to an advanced HF center for evaluation and risk assessment. In addition to the cardiopulmonary exercise test, the Heart Failure Survival Score and Seattle Heart Failure Model are common diagnostic tools used to assess the severity of HF and determine candidacy for cardiac transplantation.^5-7^

**Table 1.** Indications for Heart Transplant Waitlisting^6-7^

Indications	Contraindications
Cardiogenic shock requiring intravenous inotropes (dobutamine, milrinone, etc)Refractory cardiogenic shock requiring IABP or LVADPeak VO_2_ <10 mL/kg/minNYHA III or IV despite maximal medical or resynchronization therapyRecurrent life-threatening left ventricular arrhythmias despite use of ICD, antiarrhythmic therapy, or catheter-based ablationEnd-stage congenital heart failure without evidence of pulmonary hypertensionRefractory angina without potential medical or surgical therapeutic options	**Absolute contraindications**Irreversible liver diseaseIrreversible pulmonary parenchymal disease (or FEV_1_ <1 L/min)Irreversible pulmonary artery hypertension (PASP >60 mmHg, PVR >5 Wood units despite use of vasodilators)Clinically severe symptomatic cerebrovascular diseaseHistory of solid organ or hematologic malignancySevere irreversible multisystem disease process**Relative contraindications**Age >70 years Severe obesity (BMI >35 kg/m^2^) or cachexia Diabetes with end organ damage other than nonproliferative retinopathy or persistent poor glycemic control (HbA1c >7.5% or 58 mmol/mol) despite best effortIrreversible renal dysfunction (GFR <30 mL/min/1.73 m^2^)Severe peripheral vascular diseaseSevere cerebrovascular diseaseSevere osteoporosisAcute pulmonary embolism (within 6 to 8 weeks)Active infection (excluding LVAD-related infections)Psychological instability Substance abuse within 6 months (alcohol, cocaine, opioids, tobacco products)Lack of social support or sufficient resources to permit ongoing access to immunosuppressive medication and frequent medical follow-upInability to comply with drug therapy on multiple occasions

BMI, body mass index; FEV_1_, forced expiratory volume in the first second of expiration; GFR, glomerular filtration rate; HbA1C, hemoglobin A1c; IABP, intraaortic balloon pump; ICD, implantable cardioverter-defibrillator; LVAD, left ventricular assist device; NYHA, New York Heart Association; PASP, pulmonary artery systolic pressure; PVR, pulmonary vascular resistance; VO_2_, oxygen consumption.

**Table 2.** **United Network for Organ Sharing Heart Allocation System as of October 2018**^8^

Status	Criteria
Status 1	Venoarterial extracorporeal membrane oxygenation
	Nondischargeable, surgically implanted, nonendovascular biventricular support device
	Mechanical circulatory support with life-threatening ventricular arrhythmias
Status 2	Nondischargeable, surgically implanted, nonendovascular left ventricular assist device
	Intraaortic balloon pump
	Ventricular tachycardia or ventricular fibrillation
	Mechanical circulatory support with device malfunction/mechanical failure
	Total artificial heart, biventricular assist device, right ventricular assist device, or
	ventricular assist device for single ventricular patients
	Percutaneous endovascular mechanical circulatory support device
Status 3	Dischargeable left ventricular assist device for up to 30 days
	Multiple inotropes or single high-dose inotropes with continuous hemodynamic monitoring
	Venoarterial extracorporeal membrane oxygenation after 7 days; percutaneous endovascular circulatory device or intraaortic balloon pump after 14 days
	Nondischargeable, surgically implanted, nonendovascular left ventricular assist device after 14 days
	Mechanical circulatory support with device infection
	Mechanical circulatory support with hemolysis
	Mechanical circulatory support with pump thrombosis
	Mechanical circulatory support with right heart failure
	Mechanical circulatory support with mucosal bleeding
	Mechanical circulatory support with aortic insufficiency
Status 4	Stable left ventricular assist device candidates not using 30-day discretionary period
	Inotropes with hemodynamic monitoring
	Retransplant
	Diagnosis of congenital heart disease
	Diagnosis of ischemic heart disease with intractable angina
	Diagnosis of hypertrophic cardiomyopathy
	Diagnosis of restrictive cardiomyopathy
	Diagnosis of amyloidosis
Status 5^a^	Combined organ transplants
Status 6	All remaining active candidates
Status 7	Inactive/not transplantable

^a^Status 5 candidates may ascend to higher acuity status if indicated based on cardiac status.

## UNITED NETWORK FOR ORGAN SHARING WAITLIST

UNOS assigns all transplant candidates a status based on their severity of illness, geographic distance between the donor and recipient, length of time on the waitlist, and blood group compatibility.^8^ Prior to October 18, 2018, the highest status, 1A, was assigned to patients who were seriously ill and had an expected survival of <1 month.^8^ These patients were typically in the hospital, on mechanical ventilation, on high doses of inotropic drugs, or required an intraaortic balloon pump or percutaneous MCS devices to maintain cardiac output.^8^ Status 1B was assigned to patients who were stable on lower-dose inotropic therapy or on durable MCS. Status 2 included stable ambulatory patients not on inotropic therapy.^8^

As of October 18, 2018, this system was replaced by a new heart allocation system summarized in Table 2.^8^ Briefly, the new allocation system was designed to improve utilization of donor hearts by modifying geographical distribution to allow broader sharing of the highest status patients and to reduce waitlist mortality.^8^

## PANEL REACTIVE ANTIBODIES

While a patient is awaiting a donor heart, calculated panel reactive antibodies (cPRA) can be measured to stratify the risk of rejection posttransplant.^9^ cPRA estimates the probability that a recipient will have an unacceptable donor based on the presence of incompatible antigens in the donor pool. The higher the cPRA, the higher the likelihood of incompatibility and risk of rejection posttransplant.^9^ Therefore, patients with high cPRA have longer wait times and poorer outcomes. Populations at risk for high cPRA are those with a history of blood transfusions, pregnancy, implant of homograft materials, previous transplantation, and use of a ventricular assist device.^9^ Thresholds of cPRA acceptable for transplantation are institution-specific. Patients with cPRA above a certain threshold may be desensitized to reduce the amount of antibodies.^9^

## SURGICAL TECHNIQUES

### Orthotopic vs Heterotopic Cardiac Transplantation

In orthotopic cardiac transplantation, the recipient's heart is removed and replaced with a donor heart. This technique is the most widely used in the modern era.^10^ In heterotopic cardiac transplantation, the donor's heart is transplanted and the recipient's own heart is left in place. Although the technique is rarely used these days, the principal advantage of heterotopic transplantation was to allow the patient's native heart to assist the donor heart in cases of severe rejection or donor heart right ventricular failure.^10^

### Biatrial vs Bicaval Anastomoses

In the 1960s, Shumway and colleagues described the biatrial technique in which the donor and recipient hearts were dissected at the midatrial level.^11^ This technique was revolutionary for cardiac transplantation because it allowed surgeons to overcome the technical challenge of pulmonary venous and caval anastomoses to the atrium. This technique, however, resulted in a loss of atrial contractility, atrioventricular discordance, and regurgitation.^12^

In 1990, Yacoub et al described the bicaval technique in which the left atrial cuff on the donor heart was attached to the pulmonary venous cuff of the recipient heart.^13^ This technique allowed for preserved atrial anatomy, contractility, sinus node competence, and valvular function.

**Figure 2.**
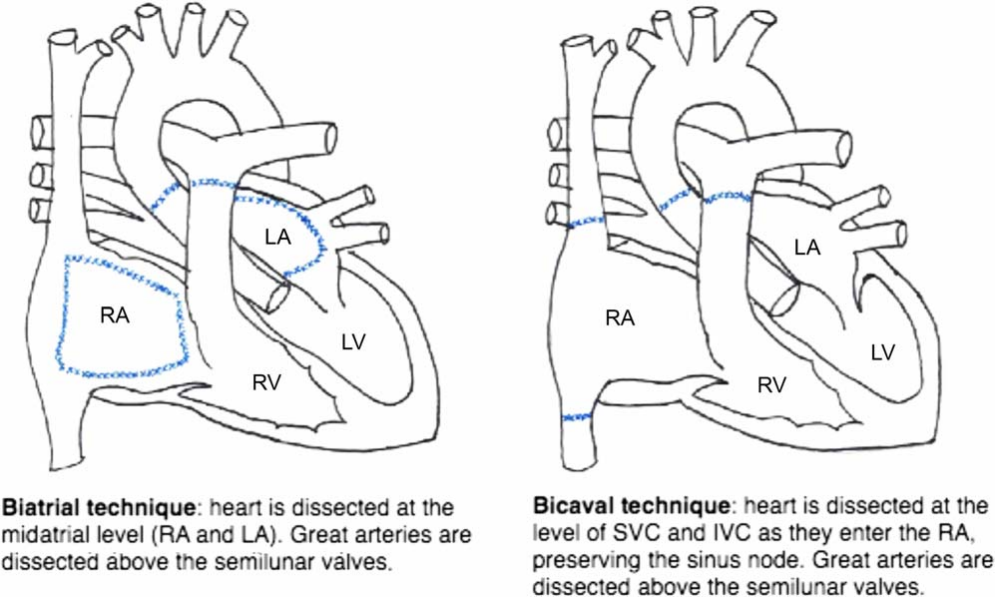
**Surgical techniques of heart transplantation.^14^** IVC, inferior vena cava; LA, left atrium; LV, left ventricle; RA, right atrium; RV, right ventricle; SVC, superior vena cava.

Figure 2 illustrates the biatrial and bicaval techniques.^14^

Studies comparing both techniques have shown that the bicaval technique reduced rates of atrial arrhythmias, atrioventricular blocks requiring pacing, tricuspid regurgitation, average length of hospital stay posttransplantation, and average mean arterial pressure at 1, 4, and 12 weeks postoperatively.^12,15^ Although the bicaval technique takes longer to complete than the biatrial technique and prolongs ischemic time, a 2010 review of 20,999 transplants noted a significant advantage in 30-day mortality in the bicaval group.^15^

## CHRONIC IMMUNOSUPPRESSION

Posttransplant, immunosuppression typically consists of a triple-therapy regimen that includes calcineurin inhibitors (CNIs), purine synthesis inhibitors, and corticosteroids.^1^ CNIs include cyclosporine and tacrolimus. Purine synthesis inhibitors include mycophenolate mofetil (MMF) and azathioprine. MMF has been shown to be superior to azathioprine in preventing rejection and mortality.^16^ Corticosteroids are usually prescribed in high doses initially with a gradual taper starting at 6 months posttransplantation.^16^

Proliferation signal inhibitors (sirolimus or everolimus) are usually added to the standard regimen (or in lieu of tacrolimus) in the setting of cardiac allograft vasculopathy or worsening renal function.^1^

Table 3 describes the most commonly used maintenance regimens.^17^

**Table 3.** Maintenance Immunosuppression Regimen^17^

Drug	Dosing	Side Effects
**Calcineurin inhibitors**		
Cyclosporine	4-8 mg/kg/day in 2 divided doses, titrated to keep target 12-hour trough levels	Renal insufficiencyHypertension and dyslipidemia
		Hypokalemia and hypomagnesemia
		Hyperuricemia
		Neurotoxicity (encephalopathy, seizures, tremors, neuropathy)
		Gingival hyperplasia
		Hirsutism
Tacrolimus	0.05-0.1 mg/kg/day in 2 divided doses, titrated to keep target 12-hour trough levels	Renal dysfunctionHypertensionHyperglycemia and diabetes mellitus
		Dyslipidemia
		Hyperkalemia
		Hypomagnesemia
		Neurotoxicity (tremors, headaches)
**Cell cycle agents**		
Azathioprine	1.5-3.0 mg/kg/day, titrated to maintain white blood cell level at approximately 3K	Bone marrow suppressionHepatitis (rare)
		Pancreatitis
		Malignancy
Mycophenolate mofetil	2,000-3,000 mg/day in 2 divided doses	Gastrointestinal (nausea, gastritis, diarrhea)Leukopenia
**Proliferation signal inhibitors**		
Sirolimus	1-3 mg/day, titrated to keep therapeutic 24-hour trough levels	Oral ulcerationsHypercholesterolemia and hypertriglyceridemia
		Poor wound healing
		Lower extremity edema
		Pulmonary toxicities (pneumonitis, alveolar hemorrhage)
		Leukopenia, anemia, and thrombocytopenia
		Pericardial effusion
		Potentiation of calcineurin inhibitor nephrotoxicity
Everolimus	1.5 mg/day in 2 divided doses	Oral ulcerations
		Hypercholesterolemia and hypertriglyceridemia
		Poor wound healing
		Lower extremity edema
		Pulmonary toxicities (pneumonitis, alveolar hemorrhage)
		Leukopenia, anemia, and thrombocytopenia
		Potentiation of calcineurin inhibitor nephrotoxicity
**Corticosteroids**		
Prednisone	1 mg/kg/day in 2 divided doses, tapered to 0.05 mg/kg/day by 6-12 months	Weight gainHypertension, hyperlipidemia, hyperglycemia
		Osteopenia
		Poor wound healing
		Salt and water retention
		Proximal myopathy
		Cataracts
		Peptic ulcer disease

## EARLY COMPLICATIONS FOLLOWING CARDIAC TRANSPLANTATION

### Primary Graft Dysfunction

Primary graft dysfunction—a syndrome in which the transplanted heart fails to meet the circulatory requirements of the recipient early in the posttransplant period—occurs in 7.4% of heart transplants and has a 30-day mortality of 30%, making it the most common cause of mortality during the first month after transplant.^18^ Risk factors for graft dysfunction include older donor age, female donor, non–head trauma as cause of death, coronary artery disease in the donor, older recipient age, prolonged ischemic time (>240 minutes), and donor-to-recipient size mismatch.^19^

When primary graft dysfunction is clinically suspected, immediate bedside echocardiography should be performed to assess for left ventricular or right ventricular failure. Treatment ranges from pharmacologic management using inotropic therapy to MCS in severe cases.^17^

### Acute Right Ventricular Failure

Acute refractory right ventricular failure occurs in 2% to 3% of patients after cardiac transplantation, although mortality is >50%.^19,20^ The etiology of right ventricular failure may be multifactorial, including prolonged ischemia-reperfusion time, elevated pulmonary vascular resistance, volume overload, inadequate preload, and donor size mismatch.^21^ Preoperative pulmonary hypertension, specifically pulmonary vascular resistance >6 Wood units and mean transpulmonary gradient >15 mmHg pretransplant, is associated with a higher incidence of right ventricular failure and perioperative mortality.^21,22^ Management of right ventricular failure and pulmonary hypertension usually includes the use of inotropes, nitric oxide, optimization of volume, and cardioversion in cases of dyssynchrony between the sinoatrial node and atrioventricular nodes.^17^ Right ventricular assist devices may be used in cases of severe right ventricular failure.^17^

### Conduction Abnormalities

Owing to denervation of the donor heart, the loss of parasympathetic efferent signals from the vagus nerve to the sinoatrial node, along with the loss of sympathetic efferent signals to the atria and ventricles, leads to an altered response of baroreceptors.^23^ In the setting of hypotension, the absence of activation of carotid and aortic baroreceptors via the sympathetic system results in an inadequate increase in cardiac output that in turn contributes to further hemodynamic instability in the posttransplant period.^23^

Right and left bundle branch blocks may also result from surgical trauma to the conduction system or prolonged ischemic time.^23^ Epicardial pacing is commonly used intraoperatively at the time of heart transplantation to maintain cardiac output with heart rates between 100 and 120 bpm. Chronotropic agents may be used to further augment heart rate. In cases of persistent bradycardia, permanent pacing can be considered.^17^

### Other Common Postoperative Complications

A retrospective study of 239 patients after heart transplantation identified the most common nonfatal complications to be pericardial effusion (61.5%), arrhythmias (41.8%), and mediastinal bleeds (8.4%).^24^ Pericardial effusion can be diagnosed clinically and confirmed by echocardiogram. An effusion that is hemodynamically stable usually does not need intervention. While vascular trauma may result from surgical incisions, the use of cardiopulmonary bypass intraoperatively may cause a decrease in coagulation factors and platelets and therefore contribute to bleeding risk.^24,25^ In addition, hypothermia and intraoperative use of heparin exacerbate abnormal hemostasis.^24,25^ Conservative management of acute blood loss anemia includes conducting interval checks of blood count and transfusing compatible leukocyte-reduced packed red blood cells. Platelets should be avoided except in cases of excessive bleeding.^17^ Mediastinal bleeds require a cardiothoracic surgery consultation and evaluation for intervention, including repair of tear or muscle flap.^17,24,25^

## CARDIAC REJECTION

Three types of rejection can occur posttransplant: hyperacute rejection, acute cellular rejection, and antibody-mediated rejection. Hyperacute rejection occurs intraoperatively immediately after the aortic cross-clamp is removed and the donor heart is exposed to the recipient's red blood cells. Because of cross-matching of blood type and panel reactive antibodies, hyperacute rejection is now uncommon.

Acute cellular rejection is a T cell reaction that occurs in 20% to 40% of patients, most commonly during the first 12 months posttransplant.^26^ Acute cellular rejection is mediated by the recipient's T cells recognizing the donor's human leukocyte antigen (HLA) molecules and is characterized by an inflammatory infiltrate on endomyocardial biopsy (EMB).^26^ Acute cellular rejection is classified based on the severity of lymphocytic infiltrate and myocyte damage (grades 0 to 3); treatment typically includes high-dose corticosteroids.^17^ If the patient has hemodynamic instability or shows no improvement 12 to 24 hours after steroid administration, antithymocyte antibodies should be used.^17^ A repeat EMB is usually performed 1 to 2 weeks after treatment for follow-up.^17^

Antibody-mediated rejection is seen in 10% to 20% of patients and has a mortality rate of 8%.^27,28^ In antibody-mediated rejection, donor antigens and recipient antibodies form an antigen-antibody complex, or a membrane attack complex, that results in endothelial and vascular injury. The diagnosis of antibody-mediated rejection is confirmed by the presence of circulating donor-specific antibodies and via EMB with immunopathologic evidence of complement activation (C4d, C3d, CD68). Management of antibody-mediated rejection includes intravenous immunoglobulin, plasmapheresis, antilymphocyte antibodies, and high-dose corticosteroids.^17^ The maintenance immunosuppression regimen is typically optimized in the setting of antibody-mediated rejection, and rituximab may be added as a second-line agent to reduce the risk of recurrent rejection.^17^

While EMB is the gold standard screening test for rejection, it is an invasive procedure that has associated risks.^17^ AlloMap (CareDx) is now an approved noninvasive screening test for acute cellular rejection in low-risk patients.^29^ Using polymerase chain reaction, this test measures the expression of 20 genes and generates a score ranging from 0 to 40. Scores <34 have been associated with a low likelihood of moderate to severe cardiac allograft rejection.^29^ This test is currently widely used and has helped transplant centers reduce the number of EMBs needed after cardiac transplantation.^29,30^

## LATE COMPLICATIONS FOLLOWING CARDIAC TRANSPLANTATION

Long-term outcomes for cardiac transplant recipients remain suboptimal, with a median cardiac allograft survival of 11 years.^31^ Long-term posttransplant complications include chronic allograft vasculopathy, increased risk of malignancies, opportunistic infections, and renal insufficiency.^31^

### Chronic Allograft Vasculopathy

Chronic allograft vasculopathy is accelerated atherosclerosis of blood vessels after cardiac transplantation and has an estimated incidence of 8% in the first year, 20% at 3 years, 30% at 5 years, and >50% at 10 years.^31^ Risk factors include older donor and recipient age, history of diabetes mellitus, hypertension, mismatch of body size, and mismatch of HLA.^32^ Because the donor heart is denervated during transplantation, cardiac transplant patients do not have typical anginal pain but rather atypical clinical presentations such as HF, arrhythmias, or sudden cardiac death.^32^ The International Society for Heart and Lung Transplantation (ISHLT) recommends the use of intravascular ultrasound in conjunction with coronary angiogram 4 to 6 weeks after transplant followed by annual or biannual intervals to detect chronic allograft vasculopathy.^31^ Noninvasive testing with dobutamine stress echocardiogram may be done initially at 6 months posttransplant for baseline quantification of cardiac chamber sizes, pulmonary artery pressure, and right and left ventricular function and can be repeated to lengthen the interval time between angiographic screenings. Cardiac magnetic resonance and positron emission tomography tests have diagnostic accuracy for early chronic allograft vasculopathy detection.^32,33^ Gene profiling has also emerged as a promising noninvasive technique to detect chronic allograft vasculopathy.^32,33^

If chronic allograft vasculopathy is localized, stenting with percutaneous coronary intervention can be attempted. If diffused, treatment consists of modifying immunosuppression, considering the use of everolimus (a drug that has been shown to reduce the incidence of chronic allograft vasculopathy),^34^ and increasing the statin dose. Retransplantation may be considered for patients who develop chronic allograft vasculopathy refractory to medical and interventional therapy and have symptoms of HF or ischemia.^17^

### Infection

Infection is a common complication in the acute and chronic setting posttransplant, accounting for 30% mortality during the first year followed by a decline to 10% to 13% per year.^1^ The predominant infections during the first month and during the first 6 months are bacterial septicemia and pulmonary infections, respectively.^35^ A viral infection, especially cytomegalovirus (CMV), may predispose a patient to a bacterial infection.^35^ Seventy-five percent of pneumonia cases occur during the first 3 months posttransplant, with most causal agents being opportunistic (60%) and nosocomial (25%), namely CMV, *Aspergillus* species, and *Pneumocystis jirovecii*.^35^ Typically, preventive vaccinations against pneumococcal pneumonia and influenza are administered prior to transplant. Posttransplant, prophylactic therapy for *Pneumocystis jirovecii* pneumonia, herpes simplex virus, toxoplasmosis, and oral candidiasis should be started. Recipients of CMV-positive hearts who are inherently CMV-negative must also receive prophylactic antiviral therapy.^17^

### Malignancy

Malignancies are a risk post cardiac transplantation, specifically skin cancer (18%) and lymphoma (1.9%).^1^ Patients should visit the dermatologist yearly for evaluation of any suspicious lesion and receive age-appropriate cancer screening from their primary care physician.^17^ While reduction of steroid dose in the setting of malignancy is controversial, use of proliferation signal inhibitors, such as sirolimus, may be considered.^36^

### Chronic Kidney Disease

Chronic kidney disease (CKD) is common in up to 50% of patients 5 years after heart transplantation, with 6% requiring dialysis by 10 years posttransplant.^36^ Risk factors include older age, female sex, lower pretransplant glomerular filtration rate (GFR), and pretransplant inotrope or MCS use.^37^ CNIs, a mainstay of immunosuppression after cardiac transplantation, contribute to a yearly risk of progressive CKD and overall mortality 5 years posttransplant.^36^ To prevent progression, renal function must be monitored regularly, renal toxins should be minimized, and the CNI dose should be reduced. Referral to a nephrologist should be considered if GFR falls below 30 mL/min/1.73 m^2^ or in cases of significant proteinuria (>500 mg/d).^17^ Strict glucose and blood pressure control helps slow progression of CKD. Hemoglobin should be measured once yearly in all cardiac transplant recipients with CKD with the goal of maintaining levels of 11 to 13 g/dL.^17^

Because CNIs have a significant effect on progressive renal disease, a randomized controlled trial evaluated whether substituting everolimus for a CNI 7 to 11 weeks after cardiac transplantation affected renal function.^38^ The study noted a protective effect on the GFR measured 12 and 36 months posttransplant in the group that was switched from a CNI to everolimus while maintaining other immunosuppression with mycophenolic acid and corticosteroids.^38^ However, this group also had higher rates of biopsy-proven acute rejection episodes and serious adverse events at 36 months posttransplant.^38^ Therefore, early withdrawal of CNIs may only be an option for carefully selected cardiac transplantation recipients with severe renal function while acknowledging the potential for rejection.

### Other Steroid-Related Complications

Corticosteroids are a mainstay of immunosuppression after cardiac transplantation and are associated with a number of steroid-related comorbidities, including bone disease, diabetes, hypertension, and hyperlipidemia. Steroid-induced bone disease is prevalent in 25% to 50% of patients post cardiac transplantation and is associated with a 10% to 36% increased incidence of fractures.^39^ All cardiac transplant recipients should be screened for preexisting bone disease prior to transplant and prescribed calcium and vitamin D supplements to maintain vitamin D levels >30 ng/mL.^17^ ISHLT recommends taking bisphosphonates to minimize bone resorption.^17^ While steroid-induced diabetes may be prevalent in 23% of patients during the first year after cardiac transplantation, early withdrawal of steroids after transplantation may result in a decreased prevalence of diabetes therafter.^40^ Steroid withdrawal can be achieved in 50% to 80% of patients without an increase in rejection-related mortality.^40^ In addition, patients weaned off steroids and those who are maintained on low-dose maintenance corticosteroid therapy may have a decreased prevalence of comorbidities, as well as decreased incidence and progression of cardiac allograft vasculopathy.^40,41^

## CURRENT CHALLENGES AND FUTURE DIRECTIONS

Cardiac transplantation has progressed greatly since the first successful operation in 1967.^2^ However, many challenges still face physicians and heart transplant recipients.

The most critical issue is the growing number of patients on the waitlist and the relatively stagnant donor pool.^42^ The hope is that the new UNOS heart allocation system will allow previously disadvantaged patients such as patients with congenital heart disease, infiltrative cardiomyopathy, and refractory arrhythmias to obtain transplants in a timely fashion.^43,44^

In addition to optimizing the heart allocation system, further advances are needed in monitoring and preventing rejection of transplanted organs.^45^ The current gold standard for monitoring rejection in recipients is right ventricle EMB, an invasive procedure that has risks. However, research from 2010 suggests that gene expression profiling is noninferior to EMB in assessing the composite primary outcome of rejection.^46^ While the results of this study suggest a less-invasive alternative to routine biopsy for monitoring rejection in patients 6 months after transplant, further comparative studies are necessary before the relative safety of this method as an alternative to EMB can be demonstrated.^46^

More research is necessary to fill high-priority knowledge gaps in cardiac transplantation, including waitlist mortality on the new allocation system, individualizing immunosuppression regimens, and a more complete understanding of antibody-mediated rejection.^47^

## CONCLUSION

Cardiac transplantation remains the only panacea for end-stage HF. The tremendous progress in surgical techniques and immunosuppression regimens has led to better outcomes. Nevertheless, the limited number of heart donors continues to make this therapy a road less traveled.

## Notes

This article meets the Accreditation Council for Graduate Medical Education and the American Board of Medical Specialties Maintenance of Certification competencies for Patient Care and Medical Knowledge.
